# Improvement effect of insulin resistance in one-day outpatient service by reducing stress adaptation disorders in patients with gestational diabetes mellitus

**DOI:** 10.3389/fnut.2024.1450127

**Published:** 2024-11-20

**Authors:** Yan Feng, Quan Yu, Fuqian Gu, Qi Feng, Yinghong Zhang

**Affiliations:** ^1^Department of Clinical Nutrition, Yuhuangding Hospital Affiliated to Qingdao University, Yantai, China; ^2^Department of Clinical Nutrition, Jinshan Hospital Affiliated to Fudan University, Shanghai, China; ^3^Department of Pediatrics, Yantai Penglai People’s Hospital, Yantai, China; ^4^Department of General Surgery, No. 990 Hospital of PLA, Zhumadian, China; ^5^Department of Obstetrics, Yuhuangding Hospital Affiliated to Qingdao University, Yantai, China

**Keywords:** GDM, one-day outpatient, stress adaptation, insulin resistance, oxidative stress

## Abstract

**Aim:**

Conducted a one-day outpatient service for GDM patients, analyzed the relationship between stress adaptation disorder and insulin resistance in GDM patients after intervention, and tried to provide some new clues for the prevention and treatment of GDM, provide some theoretical basis for the multidisciplinary diagnosis and treatment model of GDM patients.

**Methods:**

240 GDM women were included in this study, 120 women were included in one-day diabetes clinic management for GDM women as GDM Intervention Group, and 120 GDM women receiving regular dietary education as GDM Control Group. One-day diabetes clinic management including disease knowledge and dietary education, sports education and blood sugar monitoring and personalized issues and follow-up visits, and intervention time lasting for 1 month.

**Results:**

After intervention, the concentration of 2-h postprandial blood glucose, and HOMA-IR were decreased in GDM Intervention Group, while weekly weight gain rate and insulin application rate were significantly lower than GDM Control Group (all *p* < 0.05). Cortisol and MDA in GDM Intervention Group were significantly lower than GDM Control Group (both *p* < 0.01). HOMA-IR was positively correlated with weight gain, E, NE and cortisol (*r* = 0.249, 0.242, 0.663, 0.313, all *p* < 0.01), E and HOMA-IR were negatively correlated with SOD in GDM Intervention Group (*r* = −0.306, −0.213, both *p* < 0.01).

**Conclusion:**

The intervention model in our study was based on the one-day outpatient comprehensive management model of diabetes, which improved the insulin resistance of GDM patients. The possible mechanism was related to the implementation of one-day outpatient intervention measures, which reduced the stress adaptation disorder and oxidative stress injury of GDM patients. At the same time, the implementation of intervention measures reduced the rate of weight gain, which can also alleviate insulin resistance to a certain extent. One-day outpatient treatment has a positive effect on improving insulin resistance in GDM women, which can reduce the risk of maternal and fetal complications.

## Introduction

The prevalence of GDM fluctuates between 5 and 25% worldwide, according to race, age, body composition, screening and diagnostic criteria (1, 2). In the United States, about 10% of pregnant women suffer from GDM, and nearly 90% of hyperglycemia that occurs during pregnancy belongs to GDM. The prevalence of GDM in Asian women was much higher than in the United States (3). In recent years, the incidence rate of gestational diabetes in China has increased year by year. According to statistics, the total incidence rate of GDM in Chinese Mainland can be as high as 14.8%, while age, weight gain and diabetes family history factors can significantly increase the incidence of GDM. China may have the highest number of GDM patients in the world (4).

The occurrence of GDM can also lead to adverse maternal and fetal outcomes, including short-term and long-term health effects, such as premature birth, ces section, macrosomia, neonatal hyperinsulinemia, hypoglycemia, hyperbilirubinemia, and so on (5, 6). Many GDM patients have type 2 diabetes or impaired glucose tolerance after delivery. Compared with normal pregnant women, the probability of postpartum type 2 diabetes in GDM patients is 7 times higher, and both mothers and infants are more likely to have type 2 diabetes, which will increase the incidence of adverse fetal outcomes and obesity in childhood, adolescence and even adulthood (7). Although the incidence rate of GDM increases, the prevention and treatment measures were limited. One day outpatient service for diabetes was one of the effective prevention and treatment measures. It can effectively control the blood sugar index of GDM patients by providing scientific diet education and exercise therapy for GDM patients, but the mechanism of effective sugar control was still unclear (8).

Our previous research found that oxidative stress injury in GDM patients was increased, and there was a mild stress adaptation disorder (9). Can one-day outpatient service improve blood glucose related indicators by improving stress adaptation disorder? In this study, we conducted a one-day outpatient clinic for GDM patients, analyzed the relationship between stress adaptation disorder and insulin resistance in GDM patients after intervention, and tried to provide some new clues for the prevention and treatment of GDM, and initially clarify the pathogenesis and prevention mechanism of glucose related diseases. At the same time, provide some theoretical basis for the multidisciplinary diagnosis and treatment model of GDM patients.

## Research design and methods

### Subjects

After fasting for 8–10 h overnight, all pregnant women will sit in the hospital lounge for 30 min at 8:00 am, collect all venous blood at 8:30 am, and then undergo 75 g OGTT to screen for GDM women. Diagnosis of GDM was according to the American Diabetes Association criteria with a 75-g oral glucose tolerance test (OGTT) at 24–28 weeks of pregnancy, with the cutoff value being >5.1 mmol/L at fasting, >10.0 mmol/L at 1 h, >8.5 mmol/L at 2 h (10). 240 pregnant women with confirmed GDM were included in this study at 24–28 weeks.

Diagnosed GDM patients were divided into two groups. One group was the GDM Control Group. Obstetricians will recommend patients to visit an obstetric nutrition clinic, provide basic dietary advice, guide blood glucose monitoring, and require regular follow-up visits.

The other group was the GDM Intervention Group, mainly based on a comprehensive management model of 1 day outpatient service. Except as required by the GDM Control Group, patients voluntarily participate (due to fees). One day outpatient course per week, with 10 participants included each time. We included a total of 120 patients with gestational diabetes in 1 day outpatient.

The members of the one-day outpatient clinic mainly include obstetricians, obstetric nurses, clinical nutritionists, patients, and their families. At the same time, professional health education tools, specialized food models, blood glucose meters, etc. were prepared at the pregnant women’s school.

**Figure 1:**
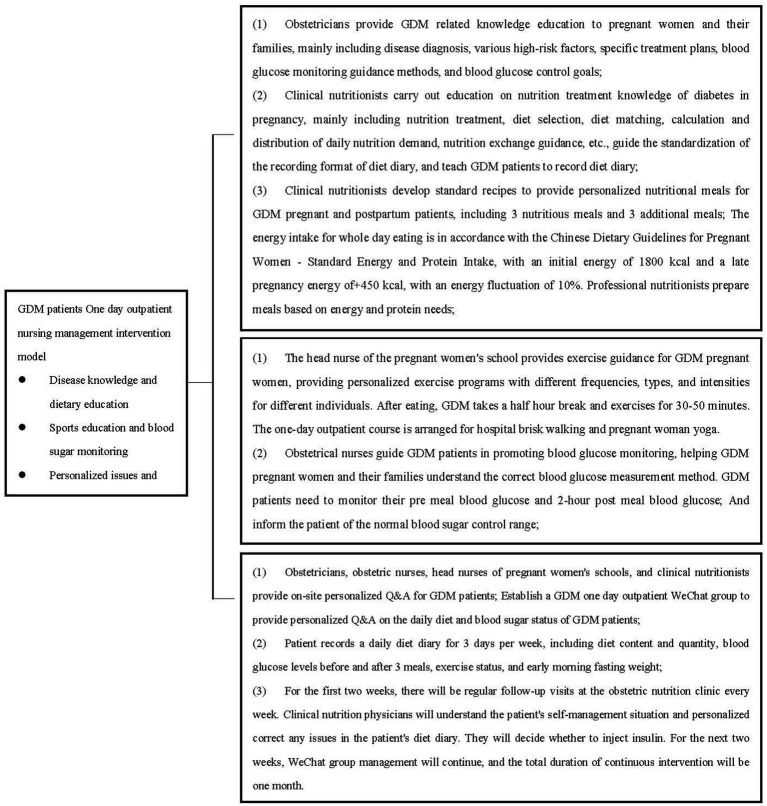
One day outpatient nursing management intervention model for diabetes.

One day outpatient nursing management intervention model for diabetes:

GDM pregnant and postpartum patients and their families will receive comprehensive centralized management interventions for a one-day outpatient clinic (Figure 1).

*Inclusion criteria*:

1. Age range from 20 to 34 years old;
2. Pregnancy 24–28 weeks;
3. Single tire;
4. Fasting plasma glucose (FPG) < 6.1 mmol/L before ambiguity

*Exclusion criteria*:

1. Pregnant women with type 1 diabetes, type 2 diabetes, hypothyroidism, hyperthyroidism and other diseases in the past;
2. Pregnant women with a history of familial genetic predispositions such as thyroid disease, coagulation dysfunction, and hypertension;
3. Pregnant women with a history of using hormones or other medications that affect blood glucose;
4. Pregnant women with mental or expression disorders;
5. Pregnant women with autoimmune diseases or infectious diseases;
6. Pregnant women who have special circumstances during pregnancy and were unable to exercise;

Approval was obtained from Ethics committee of Yantai yuhuangding Hospital, and informed consents were obtained from all participants.

#### Characteristics of GDM women

Maternal age, pre-gestational body mass index (BMI), gestational weeks and weight gain were included in clinical features of GDM women. Pre-BMI = pre-gestational weight (kg)/ height (m^2^).

#### Blood glucose, stress hormones, oxidative stress injury markers and HOMA-IR in GDM women

Implement corresponding intervention measures for GDM women who meet the conditions. After the intervention was completed, all pregnant women will receive fasting venous blood and 2-h venous blood in the morning. Fasting venous blood was used for the detection of fasting blood glucose, insulin, glycated hemoglobin (HbA1c), stress hormones, and oxidative stress indicators; The 2 h serum was mainly used to detect 2 h blood glucose.

Glucose oxidase method was used to measure fasting, 1 h and 2 h blood glucose, and they were detected by Roche automatic biochemical analyzer (Roche Diagnostics, Mannheim, Germany), and fasting insulin levels and cortisol were tested by electrochemical luminescence immunoassay (Roche Diagnostics, Mannheim, Germany); Epinephrine (E) and noradrenaline (NE) levels in GDM women were measured by radioimmunoassay method, all of which were Roche’s matching reagents; Homeostatic model assessment of insulin resistance (HOMA-IR) formula was used to assessed the degree of insulin resistance (IR). HOMA-IR = Fins (mU/ L) × FPG (mmol / L) / 22.5 (11).

Malondialdehyde (MDA) and superoxide dismutase (SOD) were used as markers of oxidative stress injury. They were detected by their corresponding reagent kits according to the instructions (Jiancheng Bioengineering Institute, Nanjing, China). Results were expressed as umol/L, U/ml. Intervention time was 1 month, weekly weight gain (kg) after intervention = weight gain (kg)[After intervention weight - pre intervention weight]/4.

### Statistical analysis

SPSS 21.0 statistical analysis software (SPSS, Inc., Chicago, IL, United States) was used for database management and statistical analysis. Data was represented by mean ± standard error. The mean comparison between two groups can be analyzed using one-way ANOVA after passing the normal test and homogeneity of variance test. Data of HOMA-IR was not normal, and it was compared after logarithmic transformation. Analysis of insulin usage rate between two groups using chi square test. The correlation between stress hormones, oxidative stress, and HOMA-IR were analyzed by multiple linear regression analysis. Data were checked for normality before analysis by using Kolmogorov–Smirnov (K-S) test. All hypothesis tests were performed bilaterally. *p* < 0.05 was considered statistically significant.

## Results

### Maternal characteristics of GDM control and GDM intervention group women

The maternal characteristics including the maternal age and gestational age at screening were similar in GDM control and GDM Intervention Group at entry to the study. Pre-gestational BMI in GDM Control Group was 0.32% higher than that in GDM Intervention Group, Weight gain in in GDM Control Group was 5.96% higher than that in GDM Intervention Group (both *p* > 0.05; Table 1).

**Table 1:** Maternal characteristics in Control and GDM Intervention Group women ( $\bar{x}$ ± s).

	GDM control group (*n* = 120)	GDM intervention Group (*n* = 120)	F	*p*
Maternal age(years)	32.27 ± 4.098	32.18 ± 4.315	0.42	0.838
Gestational age at screening(weeks)	27.03 ± 2.35	27.33 ± 2.43	1.334	0.249
Pre-gestational weight	65.41 ± 10.95	66.46 ± 13.54	0.654	0.419
Pre-gestational BMI(Kg/m^2^)	24.86 ± 4.84	24.78 ± 3.79	0.036	0.850
Weight gain(kg)	9.23 ± 4.70	8.68 ± 3.55	2.176	0.141

Values are expressed as mean ± standard error.

Comparison of weight gain, blood glucose indexes and HOMA-IR in GDM women before and after intervention.

### Before intervention

There was no significant difference between the two groups in fasting blood glucose, 1-h postprandial blood glucose, 2-h postprandial blood glucose, HbA1C(%), HOMA-IR and weekly weight gain rate (all *p* > 0.05; Table 2).

**Table 2:** Comparison of weight gain, blood glucose indexes and HOMA-IR in GDM women.

		GDM control group	GDM intervention group	F	*p*
Before intervention	Fasting blood glucose(mmol/L)	5.41 ± 0.87	5.43 ± 0.71	0.24	0.877
	Postprandial blood glucose at 1 hour(mmol/L)	8.26 ± 0.97	8.22 ± 0.72	0.191	0.663
	Postprandial blood glucose at 2 hour(mmol/L)	7.26 ± 096	7.19 ± 0.70	0.724	0.395
	Weight gain(kg/w)	0.41 ± 0.67	0.44 ± 0.50	2.176	0.141
After intervention	Fasting blood glucose(mmol/L)	5.69 ± 1.41	5.72 ± 0.92	1.564	0.212
	Postprandial blood glucose at 2 hour(mmol/L)	7.41 ± 1.34	6.75 ± 1.12^*^	3.976	0.047
	Fasting insulin	19.70 ± 8.90	16.58 ± 7.46^**^	12.881	0.000
	HbA1C(%)	5.48 ± 0.43	5.46 ± 0.40	0.198	0.656
	HOMA-IR	4.74 ± 2.32	3.96 ± 1.82^**^	12.564	0.000
	Weight gain (kg/w)	0.23 ± 0.07	0.17 ± 0.11^*^	4.376	0.042

Values are expressed as mean ± standard error.

*indicate *P* < 0.05 vs. Control Group.

**indicate *P* < 0.01 vs. Control Group.

### After intervention

Compared with GDM Control Group, the concentration of 2-h postprandial blood glucose, fasting insulin and HOMA-IR were decreased by 8.91, 15.84 and 16.46%, respectively, in GDM Intervention Group (*p* < 0.05, <0.01, <0.01). In GDM Intervention Group, weekly weight gain rate was 26.09% lower than GDM Control Group (*p* < 0.05).

HbA1C level in GDM Intervention Group was 1.09% lower than that GDM Control Group, but the difference was not statistically significant (*p* > 0.05), and there was no statistically significant difference in fasting blood glucose between two groups (*p* > 0.05).

The insulin application rate in the GDM Intervention Group (1/120) was significantly lower than that in the GDM Control Group (5/120; *p* < 0.05).

### Stress hormones and oxidative stress injury in GDM intervention group

There was no significant difference between the two groups in stress hormones and oxidative stress injury indexes before intervention (all *p* > 0.05; Table 3).

**Table 3:** Stress hormones and oxidative stress injury in Control and GDM women ( $\bar{x}$ ± s).

		GDM control group	GDM intervention group	F	*p*
Before intervention	E(ng/L)	211.23 ± 79.33	249.23 ± 121.33	0.069	0.818
	NE (ng/L)	143.88 ± 68.29	167.24 ± 92.42	0.041	0.858
	Cortisol(nmol/L)	298.34 ± 92.11	341.43 ± 126.19	0.076	0.809
	MDA(umol/L)	7.38 ± 3.28	6.39 ± 3.39	0.044	0.853
	SOD(U/mL)	122.19 ± 45.66	145.12 ± 76.30	0.067	0.821
After intervention	E(ng/L)	248.51 ± 93.64	219.15 ± 100.75**	8.085	0.005
	NE (ng/L)	170.76 ± 78.72	167.34 ± 78.59	0.168	0.682
	Cortisol(nmol/L)	336.72 ± 127.41	300.68 ± 112.62**	8.014	0.005
	MDA(umol/L)	10.35 ± 3.76	8.57 ± 3.18**	23.363	0.000
	SOD(U/mL)	163.19 ± 66.55	146.42 ± 38.94**	8.532	0.004

Values are expressed as mean ± standard error. E, Epinephrine; NE, Noradrenaline; MDA, Malondialdehyde; SOD, Superoxide dismutase; GSH, Glutathione.

*indicate *P* < 0.05 vs. Control Group.

**indicate *P* < 0.01 vs. Control Group.

The content of E and cortisol in GDM Intervention Group were decreased by 11.81 and 10.70%, respectively, as compared with GDM Control Group (both *p* < 0.01).

The same trend was observed in NE, but the difference was not statistically significant (*p* > 0.05). Compared with GDM Control Group, the content of MDA and SOD in GDM Intervention Group were decreased by 17.20 and 10.28%, respectively, (both *p* < 0.01), which indicating that oxidative stress injury in GDM Intervention Group was less severe than that in GDM Control Group (Table 3).

To confirm the intervention effect, we also compared the differences between each group of patients before and after intervention. We found that, stress hormones only Cortisol in GDM Control Group is higher than GDM Intervention Group (*p* < 0.01); Oxidative stress index only MDA in GDM Control Group is higher than GDM Intervention Group (*p* < 0.01; Table 4).

**Table 4:** Correlation of stress hormones and oxidative stress with HOMA-IR.

	GDM control group (d1)	GDM intervention group (d2)	F	*p*
E	39.80 ± 13.84	37.86 ± 10.71	0.173	0.678
NE	15.02 ± 6.25	13.74 ± 7.60	1.012	0.317
Cortisol	28.87 ± 10.40	8.79 ± 16.21	65.264	0.000*
MDA	−0.99 ± 0.05	1.26 ± 0.44	160.993	0.000*
SOD	22.93 ± 15.44	16.77 ± 27.84	2.245	0.137

Values are expressed as mean ± standard error. E, Epinephrine; NE, Noradrenaline; MDA, Malondialdehyde; SOD, Superoxide dismutase; GSH, Glutathione.

*indicate *P* < 0.05 vs. Control Group.

**indicate *P* < 0.01 vs. Control Group.

The changes in stress hormone indicators and oxidative stress products in patients after intervention are smaller, and the intervention has a certain effect.

### Correlation of stress hormones and oxidative stress with HOMA-IR in GDM intervention group

In GDM Intervention Group, HOMA-IR was positively correlated with weight gain, E, NE and cortisol after intervention (*r* = 0.161, 0.806, 0.428, 0.506; *p* < 0.05, <0.01, <0.01, <0.01), and negatively correlated with SOD (*r* = −0.213, *p* < 0.01). However, no significant correlation between MDA and HOMA-IR was observed (*p* > 0.05; Table 5).

**Table 5:** Correlation of stress hormones with oxidative stress.

			Pre-gestational BMI	Weight gain after intervention	HbA1C	Stress hormones			Oxidative stress	
						E	NE	Cortisol	MDA	SOD
GDM Control Group	HOMA-IR	*r*	−0.017	0.046	0.042	0.738^**^	0.340^**^	0.600^**^	0. 313^**^	−0.134
		*p*	0.821	0.551	0.581	0.000	0.000	0.000	0.000	0.079
GDM intervention group	HOMA-IR	*r*	−0.023	−0.161^*^	0.034	0.806^**^	0.428^**^	0.0506^**^	0.074	−0.213^**^
		*p*	0.758	0.030	0.652	0.000	0.000	0.000	0.320	0.004

E, Epinephrine; NE, Noradrenaline; MDA, Malondialdehyde; SOD, Superoxide dismutase; HOMA-IR, Homeostasis model assessment for insulin resistance.

*indicate *P* < 0.05 vs. Control Group.

**indicate *P* < 0.01 vs. Control Group.

In GDM Control Group, E, NE, Cortisol and MDA showed a significant positive correlation with HOMA-IR (all *p* < 0.01). No significant correlation between SOD and HOMA-IR (*p* > 0.05; Table 5).

They were not significantly associated with HOMA-IR in terms of Pre-gestational BMI and HbA1C were observed in both groups (both *p* > 0.05; Table 5).

### Correlation of stress hormones with oxidative stress in GDM intervention group

In GDM Intervention Group, pre gestational BMI was positively correlated with MDA (*r* = 0.195, *p* < 0.01), while other hormone related indicators were not significantly correlated with MDA (*p* > 0.05); HbA1c was positively correlated with SOD (*r* = 0.158, *p* < 0.05), while E and HOMA-IR were negatively correlated with SOD (*r* = −0.306, −0.213, both *p* < 0.01; Table 6).

**Table 6:** Correlation analysis of stress hormones with oxidative stress.

			Pre-gestational BMI	Weight gain after intervention	HbA1C	Stress hormones			HOMA-IR
						E	NE	Cortisol	
GDM control group	MDA	*r*	−0.102	0.121	−0.004	0.249^**^	0.242^**^	0.663^**^	0.313^**^
		*p*	0.180	0.112	0.958	0.001	0.001	0.000	0.000
	SOD	*r*	0.009	0.080	−0.154^*^	−0.200^**^	0.517^**^	−0.158^*^	−0.134
		*p*	0.906	0.293	0.043	0.008	0.000	0.038	0.079
GDM intervention group	MDA	*r*	0.195^**^	−0.063	0.097	0.006	−0.134	0.102	0.074
		*p*	0.008	0.401	0.194	0.936	0.070	0.168	0.320
	SOD	*r*	0.000	−0.006	0.158^*^	−0.306^**^	−0.060	0.089	−0.213^**^
		*p*	0.998	0.940	0.033	0.000	0.418	0.232	0.004

E, Epinephrine; NE, Noradrenaline; MDA, Malondialdehyde; SOD, Superoxide dismutase; HOMA-IR, Homeostasis model assessment for insulin resistance.

*indicate *P* < 0.05 vs. Control Group.

**indicate *P* < 0.01 vs. Control Group.

In GDM Control Group, E, NE, HOMA-IR were positively correlated with MDA (*r* = 0.249, 0.242, 0.663, 0.313, all *p* < 0.01), other indicators were not significantly correlated with MDA (*p* > 0.05); HbA1c was negatively correlated with SOD (*r* = −0.154, *p* < 0.05), E and Cortisol were showed a significant negative correlation (*r* = −0.200, −0.158, *p* < 0.01, <0.05) and NE showed a positive correlation with SOD (*r* = 0.517, *p* < 0.01; Table 6).

In both groups, Weight gain After intervention and HbA1C were not correlated with MDA, the same trend occurred in SOD, Pre Gestational BMI and Weight gain After intervention were not correlated with SOD (both *p* > 0.05; Table 6).

## Discussion

Pregnant women were generally in a long-term and chronic stress state. This study shows that after 1 day of outpatient education for GDM patients, compared with the GDM Control Group, the blood glucose at 2 h after meal, fasting insulin and HOMA-IR concentrations in the GDM Intervention Group decreased by 8.91, 15.84 and 16.46%, respectively. This suggests that the one-day outpatient service for diabetes can reduce the postprandial blood glucose of GDM patients and improve insulin resistance by standardizing the patients’ diet and exercise habits, which was consistent with the previous research conclusions (12–14).

After GDM patients accepted the one-day outpatient service the intervention measures had a better effect on the improvement of insulin resistance of GDM patients. After intervention, the insulin usage rate in the GDM Intervention Group was (1/120) 0.83%, while the insulin usage rate in the GDM Control Group was (5/120) 4.66%, with a statistically significant difference, which means that the one-day outpatient and late intervention measures for diabetes, including regular exercise, regular follow-up visits and personalized question answering, can effectively control the blood glucose level of GDM and reduce the insulin use rate. Previous research has suggested that exercise can improve insulin resistance in GDM patients and significantly reduce the insulin use rate in pregnancy (15, 16).

HbA1C reflects the average blood glucose level in the last 2–3 months. In our study, the one-day outpatient intervention of GDM did not affect the level of glycosylated hemoglobin in GDM patients, and there was no statistically significant difference between the two groups in the level of glycosylated hemoglobin (5.42 ± 0.40, 5.48 ± 0.43). For GDM patients, in the early pregnancy, the insulin resistance of GDM pregnant women was relatively mild, but in 24–28 weeks of pregnancy, insulin resistance gradually increased, and continued to worsen later.

Furthermore, the 2017 China Diabetes Society guidelines pointed out that due to the increased risk of anemia in pregnant women in the middle and late stages of pregnancy, although most of it may be due to physiological dilution, HbA1c levels may be affected, and HbA1c levels are highly likely to be underestimated. Therefore, in GDM diagnosis, HbA1c was often had limited value. This was consistent with the changes in HbA1c levels in GDM patients reported by Cao et al. (14).

A meta-analysis suggests that for every 1 unit increase in BMI in women before pregnancy, the risk of GDM increases by 0.92% (17). Regarding the impact of weight gain during pregnancy on GDM, Gibson et al.’s study found that weight gain during pregnancy was an important factor leading to GDM, especially in women who were overweight or obese before pregnancy (18). At the same time, there was a close relationship between excessive weight gain during pregnancy and the occurrence of GDM, adverse delivery results, and the risk of adverse maternal and fetal outcomes (19–21). In our study, there was no difference in BMI between the two groups of GDM patients, but after the one-day outpatient intervention of GDM, the weekly weight gain rate of the GDM Intervention Group decreased (0.17 ± 0.11:0.23 ± 0.07) compared to the GDM Control Group, with statistical significance. The decrease in weight gain rate will also to some extent reduce the risk of adverse maternal fetal outcomes in GDM.

A meta-analysis conducted in 2002 showed that excessive weight gain during pregnancy was associated with the risk of large for gestational age (LGA), macrosomia, cesarean section, gestational hypertension, and other postpartum adverse events. Even for women with normal BMI before pregnancy, excessive weight gain during pregnancy greatly increases the risk of postpartum hemorrhage and LGA (22). Excessive or insufficient weight gain during pregnancy may have an impact on the maternal and fetal outcomes of GDM, therefore, weight gain during pregnancy for the outcomes of GDM patients (23). GDM patients themselves have a higher risk of various adverse maternal and fetal outcomes, and excessive weight gain during pregnancy will further increase this risk. The intervention of diabetes one-day clinic for the control of weight gain of GDM patients, to a certain extent, reduced the risk of adverse events in GDM.

After pregnancy, women were in a long-term chronic stress state, and GDM patients have increased oxidative stress damage and significantly increased stress hormones, indicating that GDM patients have mild stress adaptation disorders (24–26). Stress adaptation disorders and oxidative stress damage were important factors in the occurrence of insulin resistance, and were also one of the mechanisms leading to GDM. Our study found that after intervention, the levels of stress hormones Cortisol and oxidative stress index MDA are lower than GDM Control Group, which indicating that after 1 day outpatient intervention, the degree of stress adaptation disorder in GDM patients was significantly reduced, while the degree of oxidative stress damage decreased.

In GDM Intervention Group, its HOMA-IR was positively correlated with stress hormones E and NE, and negatively correlated with SOD, indicating that the stronger the patient’s stress adaptation disorder and oxidative stress damage, the more severe degree of insulin resistance. In addition, in GDM Intervention Group, all stress hormone indicators E, NE, and cortisol included were negatively correlated with SOD, while in GDM Control Group, stress hormones E and NE were positively correlated with MDA, which was consistent with our previous research results (9). This also means that as the degree of stress adaptation disorder increases, the degree of oxidative stress damage in GDM patients also increases, and both were related to the degree of insulin resistance in GDM patients (27, 28).

In the actual clinical work process, there were various factors that cause GDM, and there were many problems with the management strategy of GDM (29–32). Patients have insufficient understanding of the hazards of GDM, including both long-term and short-term hazards, were unable to systematically obtain standardized and scientific GDM dietary treatment plans, do not know what kind of exercise to choose and how long to exercise, do not have a good grasp of GDM blood glucose control standards, and do not have a complete understanding of pregnancy weight management. They were not clear about how to solve problems encountered during blood glucose control, and when insulin injections were needed. All of these were the most important issues for GDM patients, but they were also the most lacking in professional channels for access (33).

At present, the most commonly used methods of nutrition education for GDM patients in most hospitals include consultation and outpatient visits, but these education methods were often superficial. Due to time constraints, the education time was relatively short, leading to insufficient attention from GDM patients, unclear understanding of dietary intake, low compliance, low follow-up rate, poor self-management ability, and difficulty in maintaining healthy lifestyle habits in the long term, The ultimate blood glucose control was not ideal (34, 35). The intervention model in our study was based on the one-day outpatient comprehensive management model of diabetes, which improves the insulin resistance of GDM patients. The possible mechanism was related to the implementation of one-day outpatient intervention measures, which reduces the stress adaptation disorder and oxidative stress injury of GDM patients. At the same time, the implementation of intervention measures reduces the rate of weight gain, which can also alleviate insulin resistance to a certain extent. One-day outpatient treatment has a positive effect on improving insulin resistance in GDM women, which can reduce the risk of maternal and fetal complications. In the whole research process, the main reasons for the improvement of insulin resistance in GDM patients, in addition to the education content of diabetes one-day clinic itself, also included regular follow-up of pregnancy, childbirth and nutrition clinics in the later period and timely correction of personalized problems in WeChat groups.

GDM Intervention Group patients included in our study may also have a certain degree of selection bias. Because GDM patients who were willing to actively participate in a one-day outpatient clinic and regularly follow up pay more attention to their own height and blood glucose issues, and their initiative and self-management ability were higher than those of ordinary GDM patients. GDM Intervention Group was more likely to achieve the results of “lower stress adaptation disorder and lighter insulin resistance.” If we have the opportunity in the future, we plan to carry out a multi-center study or a study of a large population, so as to discover the contribution of diabetes one-day outpatient service to GDM blood glucose control and improvement of maternal and infant outcomes.

## Data Availability

The raw data supporting the conclusions of this article will be made available by the authors, without undue reservation.
